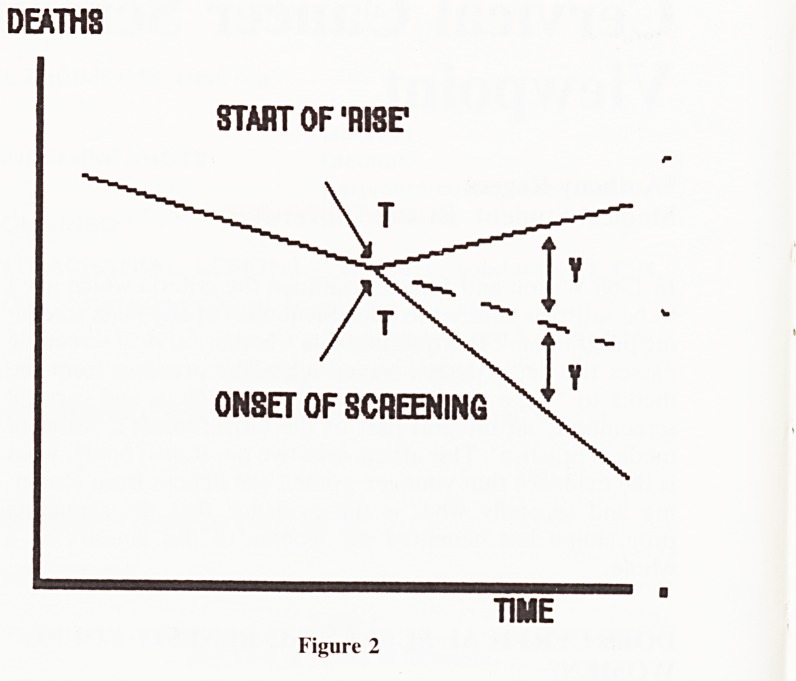# Cervical Cancer Screening–An Alternative Viewpoint

**Published:** 1990-09

**Authors:** Anthony Rogers

**Affiliations:** Medical Student, Bristol University


### Cervical Cancer Screening—An Alternative Viewpoint

*Anthony Rogers
Medical Student, Bristol University

In 1968 Wilson and Jungner outlined the criteria which need to be satisfied before the implementation of any mass screening programme[1]. It is questionable whether cervical screening passes this set of tests[2]. Nevertheless the pressure from the media to "Take the Screen Test[3]" is enormous and cervical screening is an integral part of the Government's vision of medical practice[4]. This article asks two questions; firstly, what is the evidence that younger women can benefit from screening and secondly what is the evidence that the screening programme has benefited the women of this country as a whole.

### DOES CERVICAL SCREENING BENEFIT YOUNG WOMEN?

Two of Wilson's criteria were that the disease should pose an important health problem and that its natural history should be well known[1]. Evaluation of these criteria with respect to cervical cancer in younger women will allow us to assess the threat posed by the disease and how sure we can be that our interventions will be beneficial.

Of the 4 million women aged between 15 and 24 in 1987 in England and Wales[5], 8 died from cervical cancer[6]. 1 in 60,000 women aged between 15 and 34 died from the disease[5,6]. Distressing as each individual case may be, cervical cancer in young women is not a major health problem. A nationwide screening programme cannot and should not be expected to prevent these deaths. Sensitivity is inversely proportional to specificity and so the costs, both to the tax payer and well women in the programme, would be unacceptably high. It is misleading to quote relative percentage increases in deaths and conclude that "many young women now come into high-risk groups[7]" without at the same time quoting absolute risk values. Naturally if there were evidence that the early detection and treatment of lesions in young women would protect against the development of invasive cancer later in life then the quest to find these lesions would assume more importance. However the absence of randomised controlled trials means that the natural history of cervical cancer is very poorly understood. The significance of various lesions, especially in younger women, is uncertain. "Implicit in the nomenclature of cervical intraepithelial neoplasia (CIN) is the concept of tumour progression[8]" but the evidence concerning the long term outcome of such lesions is conflicting and unreliable. Studies of the progression of CIN are plagued by selection bias, inadequate follow up, difficulties in terminology[9] and marked observer variation, even among expert histopathologists[8]. As a result it has been estimated that frank malignancy develops in anywhere between 0.17% and 70% of cases[10].

Some series suggest that regression of the different 'pre-malignant' lesions is the norm in younger women[11,12]. Carcinoma *in situ* (CIS) is far more common than invasive carcinoma and has a peak frequency many decades earlier[13,14]. This suggests that either the two conditions are unrelated or that a small amount of CIS lesions convert into invasive cancer and take a very long time to do so. If progression does occur then the Walton report concluded that CIS takes more than 10 years to become invasive and dysplasia an average of 45 years[15]. If this is the case then a couple of screens around the age of 30 should pick up almost all of the lesions which had started at an early age and would ultimately be life threatening.

A nationwide screening programme cannot and should not be expected to protect against the extremely rare, rapidly invasive subgroup of cancers and the predominance of such cancers in younger women has been questioned by Robertson *et al*[16].

### HAS CERVICAL SCREENING BENEFITED THE POPULATION?

The efficacy of cervical cancer screening in the UK will never be known for certain as a randomized controlled trial has never been carried out. Such a trial would now be unethical. Therefore we are left with two methods of evaluating the outcome of screening; case control studies and observations on the temporal relationship between the onset of screening in a region and any reduction in mortality.

### Case Control Studies

One trial of this type was carried out in North East Scotland and claimed to show that screened women were 2.3 times less likely to develop symptomatic cervical cancer than unscreened women (unmatched analysis, 95% confidence interval: 1.0–5.3)[17]. However this study was particularly susceptible to selection bias as controls were chosen retrospectively and only matched with respect to age. Several studies have shown that the likelihood of attending screening is inversely proportional to the chance of developing cervical cancer[18,19]. Consequently such a trial cannot discern what percentage of the observed difference in incidence rates, if any, was due to the screening process—it may have been due to the inherently more favourable risk profiles of attenders.

### Observations On Screening Effort and Mortality Reduction

Several studies have observed regional mortality reductions but to conclude that these were due to cervical screening is tenuous. In Scotland, following the introduction of screening in one region, incidence fell by 25% in screened women (aged 20–69) but by 39% in those women not screened (aged 70 and over)[20]. Another review documents the decreasing death rates from cervical cancer since 1968 and attributes them to screening, but fails to report that rates were falling before the onset of screening[21]. It is inconsistent to dissect out age-specific mortality reductions and say these are attributable to the screening process, while citing lack of effect in those age groups most intensively screened as a reason for more screening.

Furthermore regional observations are unsatisfactory: national programmes should produce results discernible at a national level. Figure 1. shows the death rates from cervical cancer from 1950 for all women aged over 15. As is clearly shown the onset of screening in the mid 1960's has had no discernible effect on the rate at which deaths were declining.

It seems there are three possible explanations for this finding:

* Joint winner of the Alfred Edward Aust Lawrence Prize in gynocology.

* OPCS statistics on mortality 1950–83:

| YEAR | DEATH RATE PER 100,000 |
|---|---|
| 1950–4 | 11.1 |
| 1955–9 | 10.9 |
| 1960–4 | 10.5 |
| 1965–9 | 9.9 |
| 1970–4 | 8.9 |
| 1975–9 | 8.6 |
| 1980–3 | 8.3 |

**Figure 1**

Death Rates from Cervical Cancer in England and Wales*

**Figure 2**

A Screening has been successful but the number of deaths before was underestimated and/or the number of deaths since has been overestimated.
B Screening has been successful and has curtailed a rise in the death toll.
C Screening has not been successful

The first two explanations will now be assessed to determine whether the third can be rejected.

### A Have Death Rates Been Recorded Inaccurately

There is little or no evidence to suggest that inaccurate recording of deaths rates can explain the trends shown in Fig. 1. A report in 1966 concluded that nearly all deaths from cervical cancer were registered[22] and there has been no change in the international classification of the disease which may have been confounding. Retrospective scrutinisation of Cancer registration figures in the Dundee and Angus regions suggest a confounding factor in that ideally death rates should be expressed as a percentage of the total number of cervices and not the total number of women. Hysterectomy rates have tripled in the age group 35–69 from 1963–1983, reaching about 14% in women aged 55–59[24]. One would therefore automatically expect a decrease in cervical cancer deaths if there were fewer cervices and this trend would owe nothing to screening.

### B Has an Increase in Cervical Cancer Been Averted?

The second possible explanation of figure 1 is that screening has succeeded in curtailing what would otherwise have been a rising death toll from the disease. The steady decline of cervical cancer deaths shown in Figure 1 really goes against this hypothesis—is it really tenable to suggest (Fig. 2) that the onset of screening and its intensity coincided exactly, both in time and in magnitude, with an increase in the disease, to produce no overall effect? Furthermore it is often implied that the rise in overall deaths would have been caused by more cervical cancer deaths in younger women. However in 1964 only 1.4% of cervical cancer deaths occurred in women under 35[25]—the supposed 'increase' in deaths would have had to have been truly exponential, and simultaneous with the onset of screening, to annul a decrease of, say 30% of total deaths.

The concept of cervical cancer as a sexually transmitted disease and the proposed increase in promiscuity of young women, with less barrier contraception, is often cited as a reason to expect more cervical cancer. The increase in the amount of unprotected intercourse seems reasonable but obviously accurate figures are extremely hard to obtain. However as yet the evidence for a sexually transmitted, aetiologically important agent is unconvincing. When agents varying from Herpes Simplex virus to Spirochaetes and spermatozoa to smegma were considered "proof of carcinogenesis has been found to be lacking in every case[26]". The spotlight has since turned to Human Papilloma Virus (HPV) but the epidemiological evidence implicating HPV is still "rather limited[27]". As with Herpes Simplex Virus, it has been impossible to disprove that virus preferentially infects neoplastic tissue.

Cervical cancer is associated with sexual activity[28] but to say that an increase in sexual activity would automatically result in an increase in cervical cancer is as justifiable as saying more grey hair would give more hip fractures. Associated factors are not necessarily causal.

The third possible explanation of Figure 1 is that screening for cervical cancer in the UK has been largely ineffective. A comprehensive analysis of screening effort and incidence and mortality from cervical cancer in Scotland, Wales and the 14 English Health regions for the last 20 years is consistent with the conclusion that "the screening programme has been largely unsuccessful[29]." Faced with the lack of evidence to the contrary from both case control and observational studies this conclusion must be seriously considered.

In this light the detractions of screening assume relatively more importance. At least 10,000 excision biopsies are performed each year and the number of women traumatised by abnormal smears must be an order of magnitude greater. The costs of overassessment and overtreatment are not only financial; Luesley *et al.* studied a series of 915 women who had cone biopsies[30] and they found that 13% had primary or secondary haemorrhages, 17% developed cervical stenosis and 4% subsequently suffered from abnormal pregnancies or infertility. Despite the diagnosis and treatment of tens of thousands of abnormal cervical smears over 25 years the first study of the psychosexual trauma associated with this activity was only published in 1988[31]. Campion and co-workers found that a group of 56 women reported less spontaneous interest, frequency, arousal and orgasm, with more pain and negative feelings towards sex, 6 months after the diagnosis and treatment of an abnormal cervical smear. These differences were highly statistically significant, when compared to a group of controls who were well matched for the same variables beforehand.

### CONCLUSION

In England and Wales 40,000 smears and 200 excision biopsies are undertaken for every life saved—" a grievously poor cost-benefit ratio[32]". The cost of saving a life has been estimated at £300,000[33]. The reasons for the failure of cervical screening are mainly organizational and administrative[32, 34]; in Nottingham fewer than two thirds of women received the recommended follow up[35]. However other factors may also be important. The lack of effect on mortality after the treatment of so many 'pre-malignant' conditions must cast doubt on the 'dogma of progression[36]'. The sensitivity of the test in the UK has been questioned on a day to day basis[37] as well as in the much publicized tragedies in Liverpool and Oxford. Both of these observations raise the question of whether, in the search for certain pathological entities such as CIN, other aetiologically important lesions have been overlooked.

Improvements in the programme have been promised with the new computerized recall system[38] but the gross inadequacy of our population registers for the task of screening means that improvements will be very hard to achieve[39]. Perhaps refinement is suggested after critical analysis of the case for screening in younger women. Faced with the lack of evidence that younger women will benefit from screening, even when an abnormality is detected, the amount of emotional trauma associated with each case, and the extreme rarity of cancer in this age group, is it not time to divert resources? Computer simulation programmes suggest that screening women under the age of 35 is particularly unrewarding and efficiency would be greatly improved by concentrating on older age groups[40]. The almost inverse relationship between attendance and risk of developing cancer exhorts us to spend more effort on targeting services. Given the limited resources available more frequent screening would be counterproductive to the aim of more widespread screening. The IARC Working Group estimated that reducing the screening interval from 5 to 3 years would only produce a 7% extra reduction in mortality[41].

There is an ethical dimension to screening—it is fundamentally different from other aspects of medical care in that we go out into the community with an implicit promise of net benefit. Consequently informed consent is imperative. We should inform women of their absolute risks of dying from cervical cancer, our certainty (or lack of it) that we can improve these odds and the possible risks to their physical and emotional health. Such an approach may help to redress the imbalance that exists between the women at low risk who attend cervical screening and the women who need protection from cervical cancer but have never had a smear in their lives.

### REFERENCES

1. WILSON, J. M. G. and JUNGNER, G. (1968) Principles and practice of screening for disease. Geneva, World Health Organisation.
2. McCORMICK, J. S. (1989) Cervical Smears: A Questionable Practice? *Lancet* **II**, 207–9.
3. WINN, D. Take the screen test. Cosmopolitan, April 1989, 103–4.
4. Secretaries of State for Health, Wales, Northern Ireland and Scotland. Working for Patients. London HMSO, 1989.
5. OPCS 1987 Key population vital statistics, vol 14.
6. Deaths by cause, 1987 registration. OPCS Monitor, 27th September 1988.
7. ANDREWS, F. J., LINEHAN, J. J., MELCHER, D. H. (1979) Cervical Cancer in Young Women. *Lancet* **ii**, 776–8.
8. ISMAIL, S. M., COLECLOUGH, A. B., DINNEN, J. S. *ET AL.* (1989) Observer variation in the histopathological diagnosis and grading of cervical intraepithelial neoplasia. *BMJ* **298**, 707–10.
9. BUCKLEY, C. H. *et al.* (1982) Cervical intraepithelial neoplasia. *J. Clin. Path.* **35**, 1–13.
10. ANDERSON, M. C. (1985) The pathology of cervical cancer. In Cancer of the cervix—diagnosis and treatment. In Clinics in Obstetrics and Gynaecology 12(1).
11. KINLAN, L. J., SPRIGGS, A. (1978) Women with positive cervical smears but without surgical intervention. A Follow up study. *Lancet* **ii**, 1029.
13. MARSHALL, C. E. (1968) A ten year cervical screening programme. *Lancet* **ii**, 1026–9.
14. 14. COPPLESON, C. W., BROWN, B. (1975) Observations on a model of the biology of carcinoma of the cervix; a poor fit between observation and theory. *Am. J. Obstet and Gynecol* **122**, 127–36.
15. WALTON, R. J. *et al.* (1976) Cervical screening programmes. I Epidemiology and natural history of carcinoma of the cervix. *Can. Med. Assoc. J.* **114**, 1003–12.
16. ROBERTSON, J. H. *et al.* (1988) Risk of cervical cancer associated with dyskariosis. *BMJ* **297**, 18–21.
17. MACGREGOR, J. E., MOSS, S. M., PARKIN, D. M., DAY, N. E. (1985) A case-control study of cervical cancer screening in North East Scotland. *BMJ*, **290**, 1543–6.
18. ELKIND, A. K., HARAN, D., EARDLEY, A., SPENCER, B. (1987) Computer managed cervical screening. A pilot study of non-attenders. Public Health **101**, 253–66.
19. HAYWARD, A., SHAPIRO, M. F., FREEMAN, H. E., COREY, C. R. (1988) Who gets screened for cervical and breast cancer? Results of a new national survey. *Ann. Int. Med.* **148**, 1177–81.
20. MACGREGOR, J. E. and TEPER, S. (1974) Screening for cervical cancer. *Lancet* **i**, 1221–3.
21. MACGREGOR, J. E. and TEPER, S. (1978) Mortality from carcinoma of the cervix uteri in Great Britain. *Lancet* **ii**, 774–6.
22. HEASMAN, MA *et al.* OPCS studies in medical and population subjects no. 20. London HMSO 1966.
23. DUGUID, H. L. D., DUNCAN, I. D., CURRIE, J. (1985) Screening for cervical intraepithelial neoplasia in Dundee and Angus from 1962–1981 and its relation with invasive cervical cancer. *Lancet* **ii**, 1053–6.
24. COOK, G. A. anjd DRAPER, G. J. (1984) Trends in cervical cancer and carcinoma *in situ* in Great Britain. *Br. J. Cancer* **50**, 367–75.
25. OPCS Routine Statistics, 1964.
26. Anon. Human papillomavirus and cervical cancer; a fresh look at the evidence (edit). *Lancet* 1987; **i**, 725–6.
27. MUNOZ, N., BOSCH, X., KALDOR, J. M. (1988) Does human papillomavirus cause cervical cancer? The state of the epidemiological evidence. *Br. J. Cancer* **57**, 1–5.
28. BRINTON, L. A., FRAUMENI, J. F. (1986) Epidemiology of uterine cervical cancer. *J. Chron. Dis.* **39**, 1051–6.
29. MURPHY, M. F. G., CAMPBELL, M. J., GOLDBLATT, P. O. (1987) Twenty years' screening for cervical cancer in Great Britain, 1964–84: further evidence for its ineffectiveness. *J. Epid. Comm. Health* **42**, 49–53.
30. LUESLEY, D. M., McCRUM, A., TERRY, P. B., WADE-EVANS, T., NICHOLSON, H. O., MYLOTTE, M. J., EMENS, J. M., JORDAN, J. A. (1985) Complications of cone biopsy related to dimension of the cone and the influence of prior colposcopic assessment. *Br. J. Obstet and Gynaecol* **92**, 158–64.
31. CAMBPOION, M. J., BROWN, J. R., McCANCE, D. J., ATIA, W., EDWARDS, R., CUZICK, J., SINGER, A. (1988) Psychosexual trauma of an abnormal cervical smear. *Br. J. Obstet and Gynecol*, **95**, 175–81.
32. Cancer of the Cervix: Death by incompetence. (Editorial). *The Lancet*, 1985, **ii**, 363–4.
33. ROBERTS, C. J., FARROW, S. C., CHARNY, M. C. (1985) How much can the NHS afford to spend to save a life or avoid a severe disability? *Lancet* **i**, 89–91.
34. CHAMBERLAIN, J. (1984) Failures of the cervical cytology screening programme. *BMJ* **289**, 853–4.
35. CHAMBERLAIN, J. (1984) Failures of the cervical cytology screening programme. *BMJ* **289**, 891–4.
36. SKRABANEK, P. (1988) Cervical cancer screening—Time for reapprasal. *Can. J. Public Health,* **79**, 86–9.

*Continued on p. 71*

CONTD. FROM PAGE 79
CERVICAL CANCER SCREENING – AN ALTERNATIVE VIEWPOINT.

38. CUZICK, J. (1988) Cervical screening. *Edit. BJHM* p. 265.
39. ARMSTRONG, E. A. (1989) The politics of inadequate registers. *BMJ* **299**, 73.
40. KNOX, E. G. (1976) Ages and frequencies for cervical cancer screening. *Br. J. Cancer* **34**, 444–52.
41. IARC working group on evaluation of cervical screening policies. Screening for cervical squamous cancer; duration of low risk after negative results and implications for screening policies. *BMJ* **293**, 659–64.

## Figures and Tables

**Figure 1 f1:**
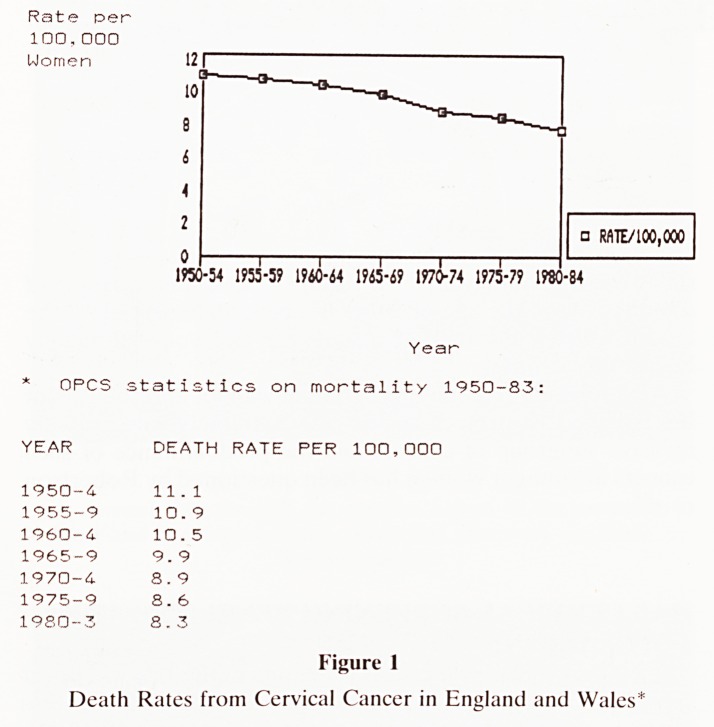


**Figure 2 f2:**